# Development and validation of a nomogram for predicting cancer-related fatigue in patients with glioma: a multicenter study

**DOI:** 10.3389/fonc.2025.1497151

**Published:** 2025-06-16

**Authors:** Qiuxia Wu, Cuiqun Su, Manxia Xing, Liangmei Ouyang

**Affiliations:** ^1^ Department of Neurosurgery, Guangdong Provincial People’s Hospital (Guangdong Academy of Medical Sciences), Southern Medical University, Guangzhou, China; ^2^ People’s Hospital of Guangdong Province Ganzhou Hospital, Ganzhou Municipal Hospital, Ganzhou, China

**Keywords:** nomogram, cancer-related fatigue, glioma, perceived social support, quality of life

## Abstract

**Background:**

Cancer-related fatigue (CRF) is one of the most prevalent symptoms which drastically affect patient health and quality of life. This study aimed to construct and validate a nomogram to accurately predict the occurrence of cancer-related fatigue in patients with glioma.

**Methods:**

This cross-sectional study included 470 glioma patients from two hospitals (training cohort: n=284; validation cohort: n=186). All patients were categorized into two groups based on their Numerical Rating Scale scores of cancer-related fatigue: a no or mild fatigue group (scores 0-3) and a moderate to severe fatigue group (scores 4-10). LASSO model and multivariable logistic regression analyses were used to determine the significant risk factors contributing to the occurrence of cancer-related fatigue in glioma patients. A nomogram was constructed and its predictive accuracy and conformity was validated by ROC curves, calibration curves and decision curve analysis.

**Results:**

Combining LASSO algorithm and multivariable logistic regression analyses, the cancer stage (p=0.014), and the scores of Perceived Social Support Scale (PSSS) (p<0.001), physical functioning (PF) (p<0.001), bodily pain (BP) (p=0.031), general health (GH) (p<0.001), and mental health (MH) (p=0.009) were the independent risk factors for cancer-related fatigue of glioma patients. A clinically quantitative predictive model nomogram was developed based on these extracted risk factors. The concordance-index of nomogram was 0.964 (0.935-0.993). The AUC values of nomogram were 0.916 (CI: 0.879-0.953) in the training cohort and 0.885 (CI: 0.829-0.941) in the validation cohort. The calibration curves of this nomogram exhibited a notable concordance with the ideal diagonal line. The decision curve analyses illuminated that this nomogram achieved high clinical net benefit.

**Conclusion:**

The nomogram, incorporating the cancer stage of glioma, perceived social support, and quality of life of patients, demonstrated good accuracy and clinical practicality. It can serve as a valuable prediction and evaluation tool for anticipating the occurrence of cancer-related fatigue in patients with glioma.

## Introduction

Glioma is the most prevalent primary intracranial tumor, representing 81% of malignant brain tumors (1). The incidence of gliomas is estimated to be around 5 to 10 cases per 100,000 people per year. Although relatively rare, gliomas can cause significant mortality and morbidity (2). The five-year survival rate for high-grade glioma is typically less than 10-15% (3). Glioma management is inherently complex. In most low-grade gliomas, the primary treatment involves maximal safe resection, which forms the foundation of therapeutic strategy (4). For patients with adult-type diffuse gliomas, the current standard of care typically involves a multimodal approach combining radiotherapy with chemotherapy, with some cases achieving remarkable long-term survival exceeding 10–20 years (5). However, avoiding surgical trauma-related harm and preventing radiotherapy- and chemotherapy-induced complications remain significant challenges (6). Additionally, the diagnostic process, treatment course, and long-term surveillance impose significant psychological distress, including clinically significant anxiety, depression, and fear of recurrence.

Cancer-related fatigue (CRF) is a debilitating and multidimensional symptom complex characterized by persistent physical exhaustion, emotional weariness, and cognitive impairment that disproportionately affects cancer patients (7, 8). Unlike general fatigue, cancer-related fatigue is more severe, persistent, overwhelming, and not easily alleviated by rest or sleep (9). People with cancer-related fatigue often describe their feelings of extreme tiredness, weakness, and weariness (10). In the majority of studies, 30% to 60% of patients report experiencing moderate to severe cancer-related fatigue during treatment, occasionally leading to the discontinuation of treatment (11). Research on long-term cancer survivors demonstrates that 25-33% of cancer survivors continue to experience chronic fatigue for up to 10 years following diagnosis (12). Cancer-related fatigue can hinder one’s ability to maintain work, social interactions, and daily routines. For some people with cancer, cancer-related fatigue may be more distressing than side effects like pain, nausea, or vomiting. It causes disruption in all aspects of life and might be a risk factor of reduced survival (13). Although the prevalence and course of fatigue in other cancer populations have been well-documented (14–16), its characteristics in glioma patients remain unknown. There is an urgent need for the development of prediction models. The proposed nomogram could offer a clinically practical tool for individualized risk prediction of cancer-related fatigue in glioma patients by visually integrating multiple prognostic factors. This modeling approach will forecast the occurrence of cancer-related fatigue in patients with glioma.

## Materials and methods

### Study participants

**Figure 1:**
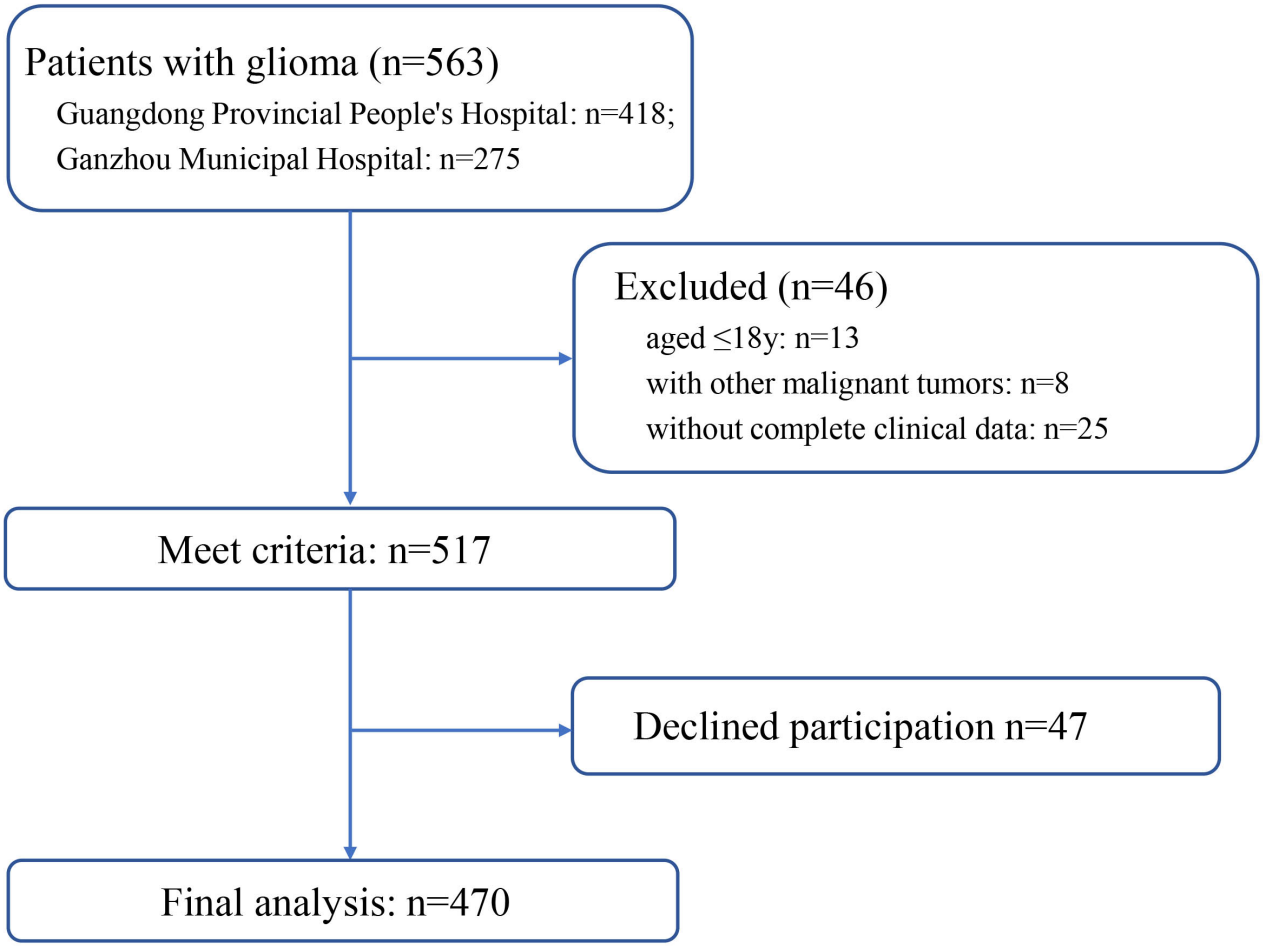
The flow diagram of patient screening process.

This cross-sectional study was conducted at the Guangdong Provincial People’s Hospital and People’s Hospital of Guangdong Province Ganzhou Hospital (Ganzhou Municipal Hospital), both of which are tertiary referral hospitals. Ethical approval for the use of clinical data for research purposes was granted by the Ethics Committee of Guangdong Provincial People’s Hospital. Between November 2022 and August 2024, we totally enrolled 470 patients from Guangdong Provincial People’s Hospital and People’s Hospital of Guangdong Province Ganzhou Hospital (Ganzhou Municipal Hospital). The flow diagram of patient screening process is shown in **Figure 1**. The study objective and data confidentiality were communicated to the participants before participating in the study. According to the declaration of Helsinki, written informed consents were acquired.

The inclusion criteria for the patients were as follows (1): age ≥ 18 years, (2) glioma confirmed through CT, MRI, and pathological examination, (3) diagnosed with glioma for the first time, (4) complete clinical data available, (5) patients with clear consciousness and literacy, capable of participating in scale assessment, (6) patients who voluntarily signed the informed consent form. Exclusion criteria were as follows: (1) psychiatric disorders, impaired mental status, or communication difficulties, (2) patients in pregnancy or lactation period, (3) concurrent diagnosis of other malignant tumors, (4) patients with acute infections or immune system disorders, (5) patients with an estimated postoperative survival time of less than 3 months, (6) history of brain inflammation or neurological deficits, (7) received psychotherapy or intervention within one year.

### Measures

The basic sociodemographic data of participant was collected using a self-prepared sociodemographic questionnaire in Chinese. The information including age, gender, body mass index (BMI), occupation, marital status, educational level, and personal monthly income. Clinical data were retrieved from the hospital information and management system, including cancer stage, chemotherapy, self-care ability, insomnia, neuroticism, pain, depression, anxiety, and physical exercise. All the information was collected one month after the patient received treatment.

In this study, the Numerical Rating Scale (NRS) was used to measure the levels of cancer-related fatigue and the intensity of pain experienced by patients with glioma (17). The NRS typically consists of a horizontal line with numbers ranging from 0 to 10. The endpoints of the scale represented the levels of cancer-related fatigue and pain, with 0 indicating no fatigue or no pain and 10 indicating the worst possible fatigue or the worst possible pain.

In order to assess sleep quality and disturbances of glioma patients over a one-month time interval, the Pittsburgh Sleep Quality Index (PSQI) was used in this study (18). Each component score of the PSQI ranges from 0 to 3, with 3 indicating the greatest dysfunction or disturbance. The seven component scores are then summed to obtain a global PSQI score, which ranges from 0 to 21. Higher scores indicate poorer sleep quality.

The Perceived Social Support Scale (PSSS) was used to measure the patients’ perceptions of the support they received from their social network (19, 20). It includes items prompting individuals to rate their agreement with statements on social support. Patients rated their responses on a Likert scale of 1 to 7 or 1 to 5, where higher scores indicating stronger perceived social support.

Health-related quality-of-life was measured by the 36-item Short Form Health Survey (SF-36) (21, 22). This is a patient-reported questionnaire that covers eight health domains: physical functioning (PF), role physical (RP), bodily pain (BP), general health (GH), vitality (VT), social functioning (SF), role emotional (RE), and mental health (MH). Different options for each item have different score weightings. The final score ranges from 0 (worst general health status) to 100 (best health status) for each domain, and higher scores indicate better quality of life.

### Risk factors extraction and analysis

In this study, all patients were categorized into two groups based on their NRS scores of cancer-related fatigue: a no or mild fatigue group (scores 0-3) and a moderate to severe fatigue group (scores 4-10). We have included 26 potential risk factors contributing to cancer related fatigue in glioma patients. There were 10 demographic and clinical factors, including age, BMI, occupation, marital status, education level, personal monthly income, cancer stage, chemotherapy, self-care ability, and physical exercise. There were 16 mental and psychological factors, including sleep quality, neuroticism, intensity of pain, depression, anxiety, perceived social support, quality-of-life, and so on.

Prior to risk factors selection, 26 potential risk factors were normalized using a z-score approach to eliminate index dimension differences of the data. In the training cohort, risk factor selection and model development were carried out. The least absolute shrinkage and selection operator (LASSO) regression with 10-fold cross-validation was employed to identify the risk factors with non-zero coefficients. Subsequently, a multivariable logistic regression analysis was performed on the risk factors identified by the LASSO model to determine significant risk factors for nomogram development.

### Nomogram construction and evaluation

A nomogram was constructed with the significant risk factors extracted by LASSO model and multivariable logistic regression analyses. The receiver operating characteristic (ROC) curve and area under the curve (AUC) illustrated the predictive accuracy and conformity of this nomogram in the training cohort and validation cohort. The C-index and calibration plots visually revealed the predictive accuracy of nomogram. Decision curve analysis (DCA) was used to evaluate the clinical net benefit of nomogram.

### Statistical analysis

All statistical analyses were performed by SPSS software version 25.0 (IBM Corporation; United States) and R software (version 3.4.1, Boston, MA, USA). The ages, BMI, and the scores of NRS, PSQI, PSSS and SF-36 were continuous variables approximately normally distributed, which were described using means and standard deviation (SD). The descriptive statistics for categorical variables were used frequencies and percentages. The differences between the training and validation cohorts in general characteristics were analyzed by Student’s t test or Pearson’s χ2 test. The multivariable logistic regression analyses were conducted to determine the hazard ratios (HR) and 95% confidence intervals (CI) for events. The variables with p<0.05 in multivariate logistic regression analyses were considered as the significant risk factors leading to cancer-related fatigue in glioma patients. We considered p<0.05 to be the statistically significance.

## Results

### General characteristics

A total of 470 glioma patients were included in this study, with 284 patients as the training cohort and 186 patients as the validation cohort. The general characteristics of two cohorts were summarized in **Table 1**. No statistical difference was observed between two cohorts, indicating the grouping was randomized and reasonable. The Numerical Rating Scale (NRS) score of cancer-related fatigue was 4.18 ± 2.77 for training cohort and 4.09 ± 2.84 for validation cohort. In the training cohort, 129 glioma patients experienced no or mild cancer-related fatigue, whereas 155 patients reported moderate or severe cancer-related fatigue. In the validation set, 104 patients were divided into the no or mild fatigue group, and 82 patients were divided into the moderate or severe fatigue group.

**Table 1:** General characteristics of patients in the training and validation cohorts.

Characteristics	Training cohort (n=286)	Validation cohort (n=184)	p value
Age (y), mean ± SD	47.47 ± 14.32	49.62 ± 14.06	0.110
BMI, mean ± SD	21.17 ± 3.11	21.05 ± 3.32	0.702
Gender			
Male	172 (60.14%)	107 (58.15%)	0.668
Female	114 (39.86%)	77 (41.85%)	
Marital status, n (%)			
Unmarried	61(21.33%)	38 (20.65%)	0.981
Married	129 (45.10%)	83 (45.11%)	
Divorced or widowed	96 (33.57%)	63 (34.24%)	
Occupation, n (%)			
Employed	187 (65.38%)	109 (59.24%)	0.178
Unemployed or retired	99 (34.62%)	75 (40.76%)	
Educational level, n (%)			
High school or lower	107 (37.41%)	64 (34.78%)	0.563
University degree or higher	179 (62.59%)	120 (65.22%)	
Monthly income (Yuan), n (%)			
Less than 5k	119 (41.61%)	82 (44.57%)	0.812
5k~10k	93 (32.52%)	56 (30.43%)	
More than 10k	74 (25.87%)	46 (25.00%)	
Clinical Stage, n (%)			
Stage I	88 (7.09%)	59 (32.07%)	0.909
Stage II	108 (48.82%)	65 (35.33%)	
Stage III	61 (22.83%)	43 (23.36%)	
Stage IV	29 (21.26%)	17 (9.24%)	
Chemotherapy, n (%)			
Yes	238 (83.22%)	160 (86.96%)	0.272
No	48 (16.78%)	24 (13.04%)	
Self-care ability, n (%)			
Yes	227 (79.37%)	139 (75.54%)	0.329
No	59 (20.63%)	45 (24.46%)	
Insomnia, n (%)			
Yes	129 (45.10%)	82(44.57%)	0.909
No	157 (54.90%)	102 (55.43%)	
Neuroticism, n (%)			
Yes	70 (24.48%)	46 (25.00%)	0.898
No	216 (75.52%)	138 (75.00%)	
Pain, n (%)			
Yes	101 (35.31%)	67 (36.41%)	0.808
No	185 (64.69%)	117 (63.59%)	
Depression, n (%)			
Yes	129 (45.10%)	87 (47.28%)	0.805
No	157 (54.90%)	97 (52.72%)	
Anxiety, n (%)			
Yes	135 (47.20%)	89 (48.37%)	0.574
No	151 (52.80%)	95 (51.63%)	
Physical exercise, n (%)			
Yes	148 (51.75%)	95 (51.63%)	0.980
No	138 (48.25%)	89 (48.37%)	
Cancer-related fatigue	4.18 ± 2.77	4.09 ± 2.84	0.751
PSQI score	8.46 ± 5.60	8.10 ± 5.61	0.493
NRS score for pain	4.24 ± 2.77	4.61 ± 2.84	0.169
PSSS score	51.14 ± 20.41	51.10 ± 20.26	0.985
Physical functioning	61.40 ± 21.76	61.73 ± 22.61	0.873
Role physical	52.54 ± 20.31	52.99 ± 21.02	0.816
Bodily pain	73.09 ± 15.82	73.08 ± 16.07	0.997
General health	65.92 ± 17.66	65.22 ± 17.76	0.677
Vitality	56.38 ± 12.75	57.47 ± 12.47	0.496
Social functioning	64.89 ± 11.50	65.95 ± 10.69	0.319
Role emotional	67.48 ± 23.27	70.65 ± 21.95	0.141
Mental health	61.11 ± 12.73	61.86 ± 12.44	0.531

### Risk factors extraction

**Figure 2:**
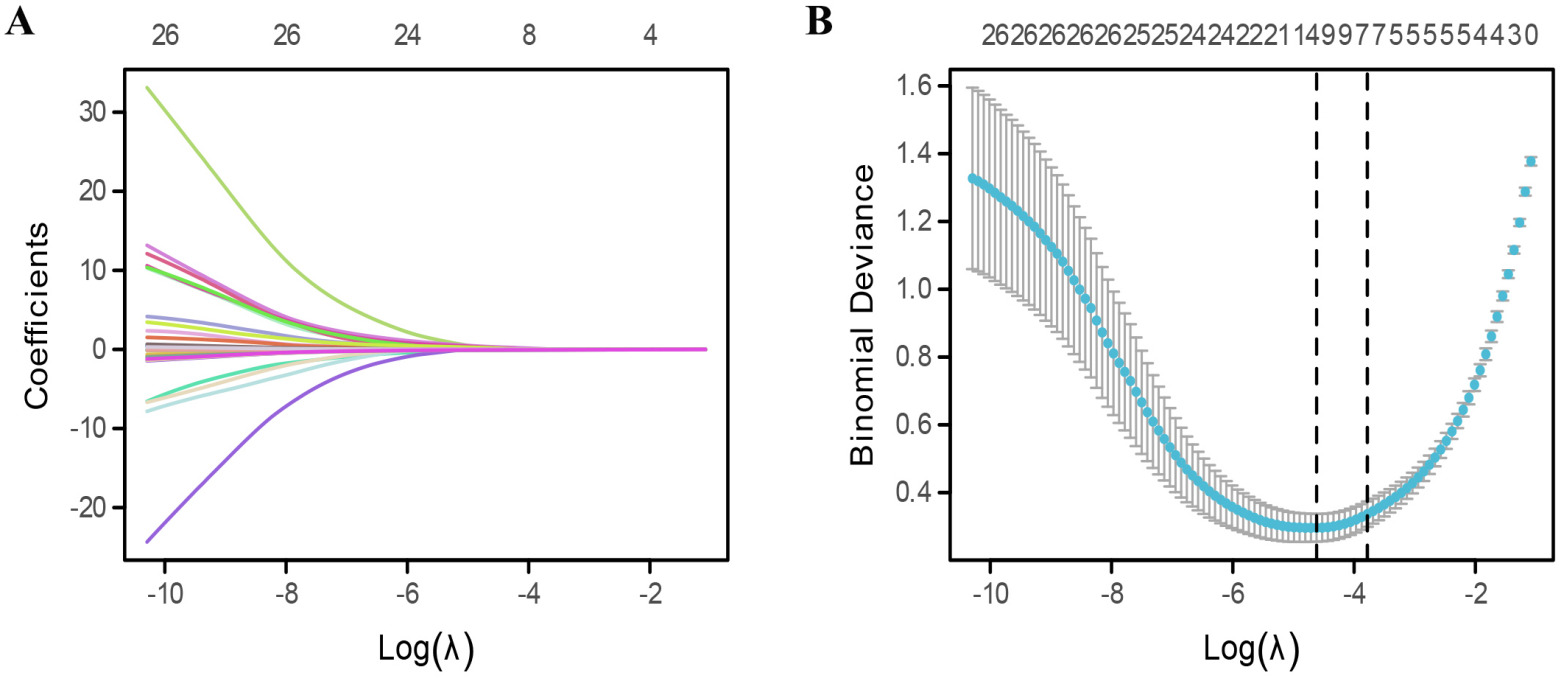
Extraction of risk factors for cancer-related fatigue. **(A, B)** Risk features extraction by using least absolute shrinkage and selection operator (LASSO) algorithm.

After LASSO algorithm, 12 risk factors were retained based on non-zero coefficients for the prediction of cancer-related fatigue (**Figure 2**). These factors included occupation, cancer stage, sleep quality, anxiety, the PSSS score, and the seven health domains of the SF-36, excluding role physical.

To determine the independent risk factors leading to cancer-related fatigue of glioma patients, multivariable logistic regression analyses were performed in the training cohort. As shown in **Table 2**, the cancer stage (p=0.014), and the scores of PSSS (p<0.001), physical functioning (PF) (p<0.001), bodily pain (BP) (p=0.031), general health (GH) (p<0.001), and mental health (MH) (p=0.009) were the independent risk factors for cancer-related fatigue of glioma patients.

**Table 2:** Multiple logistic regression analysis of risk factors of cancer-related fatigue in thyroid cancers patients.

Clinical Stage, n (%)	Odds Ratio (95% CI)	p value
Stage I	Reference	
Stage II	0.661 (0.080 - 5.453)	0701
Stage III	0.849 (0.088 - 8.166)	0.888
Stage IV	39.633 (2.101 - 747.302)	0.014
PSSS score	0.884 (0.834 - 0.938)	<0.001
Physical functioning	0.809 (0.721 - 0.907)	<0.001
Bodily pain	0.890 (0.802 - 0.989)	0.031
General health	0.820 (0.731 – 0.919)	<0.001
Mental health	0.852 (0.755 – 0.962)	0.009

### Construction and validation of nomogram

**Figure 3:**
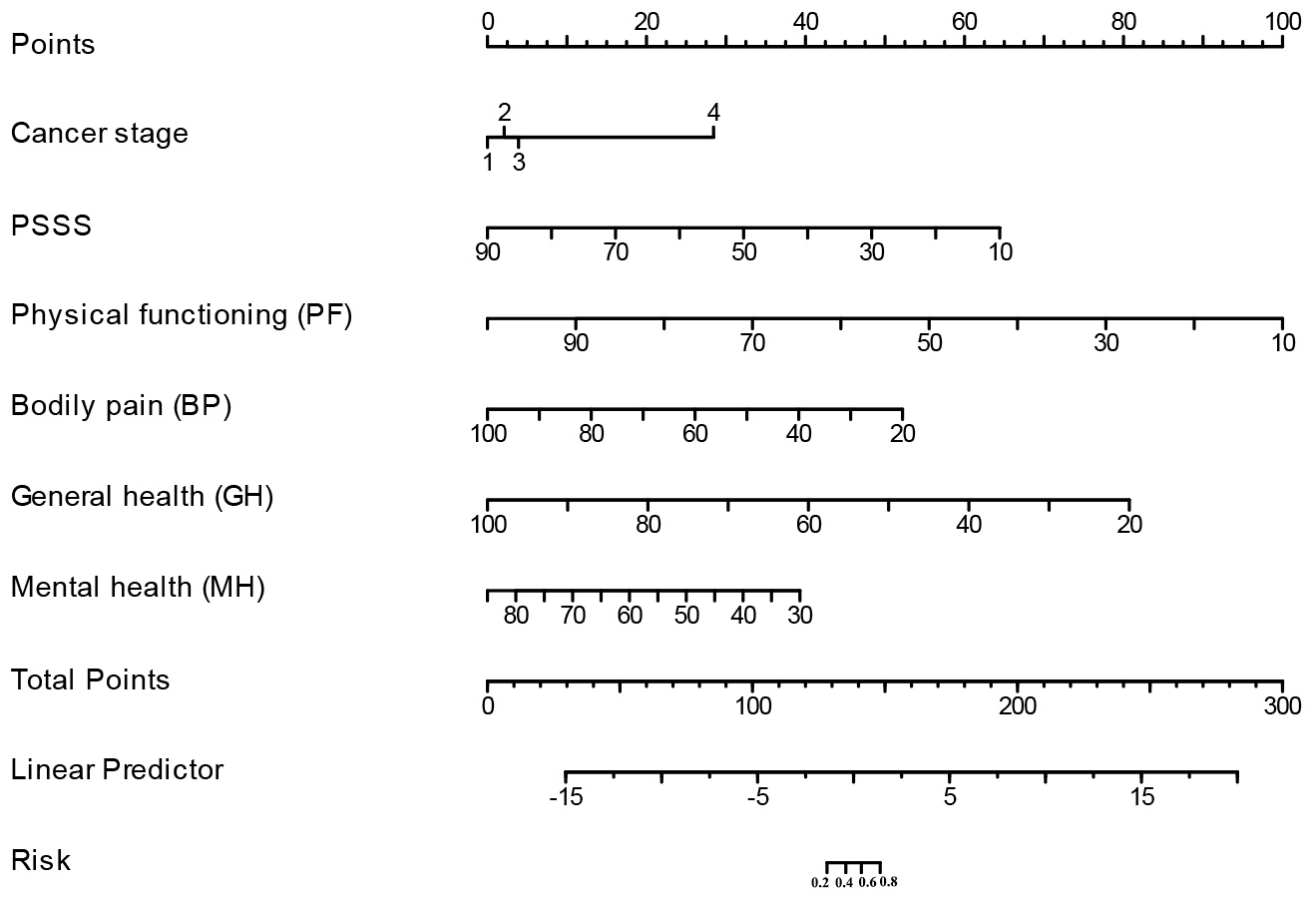
Nomogram for predicting the occurrence of cancer-related fatigue.

**Figure 4:**
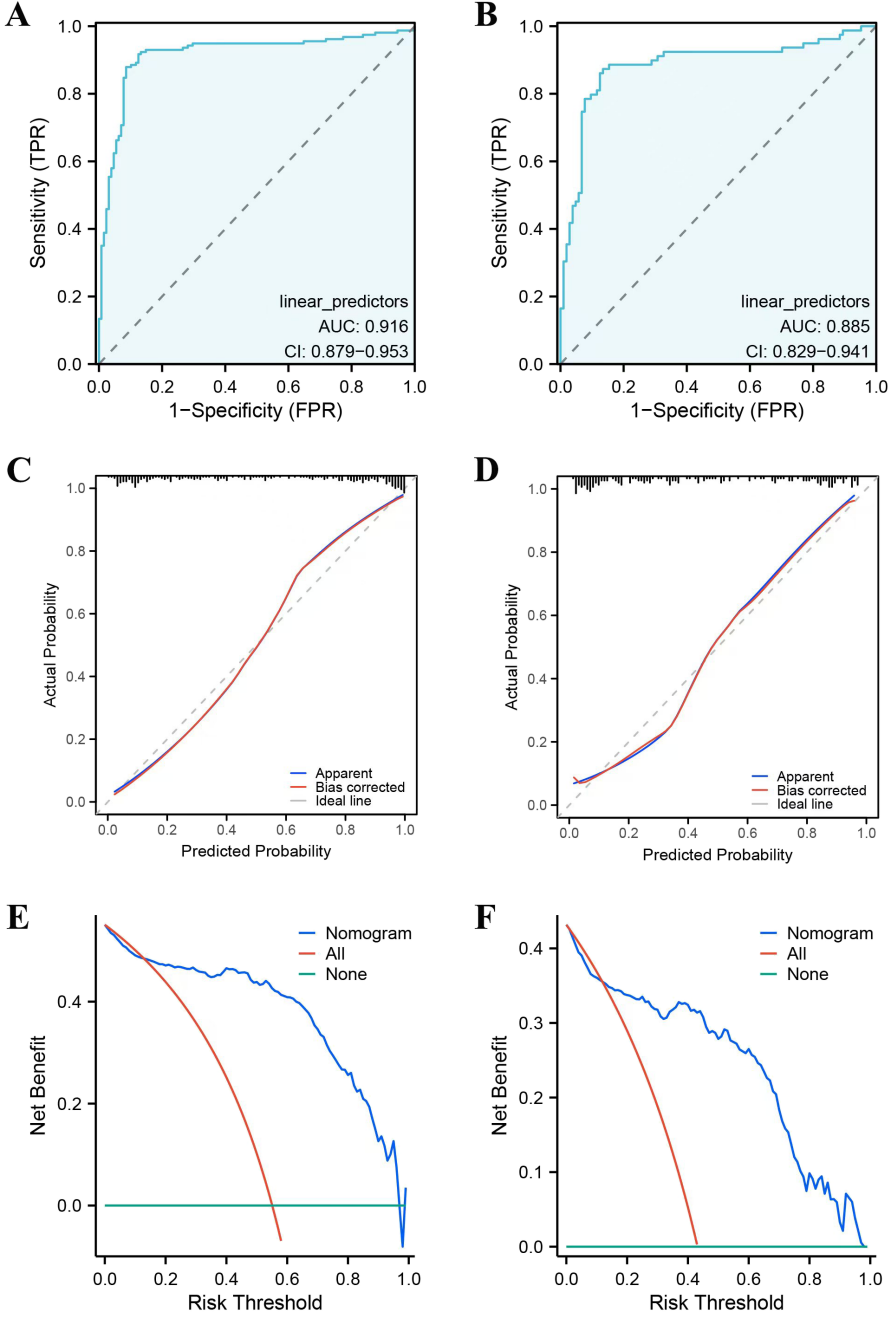
Calibration and discrimination of the nomogram. **(A)** ROC curve for training cohort. **(B)** ROC curve for validation cohort. **(C)** Calibration curve for training cohort. **(D)** Calibration curve for validation cohort. **(E)** Decision curve analysis for training cohort. **(F)** Decision curve analysis for validation cohort.

A clinically quantitative predictive model nomogram was developed based on the independent risk factors extracted above (**Figure 3**). The nomogram demonstrated excellent forecasting ability, with the concordance-index was 0.964 (0.935-0.993). For each glioma patient, higher total points indicated as a greater risk of developing cancer-related fatigue. We further examined the predictive ability of this nomogram on both the training and validation cohorts. The AUC values of nomogram were 0.916 (CI: 0.879-0.953) in the training cohort (**Figure 4A**) and 0.885 (CI: 0.829-0.941) in the validation cohort (**Figure 4B**). The calibration curves of this nomogram exhibited a notable concordance with the ideal diagonal line (**Figures 4C, D**). Moreover, the decision curve analyses (DCA) illuminated that the optimal utility of this nomogram which achieved the high clinical net benefit across the entire range of reasonable threshold probabilities in both training and validation cohorts (**Figures 4E, F**).

## Discussion

Through LASSO and multivariable logistic regression analyses, six risk factors were independently associated with cancer-related fatigue in glioma patients. To be specific, the risk factors were the cancer stage of glioma, perceived social support, and quality-of-life of patients. The nomogram model incorporating these risk factors showed favorable discrimination in predicting the likelihood of cancer-related fatigue in glioma patients. This predictive tool provides a dependable quantitative evaluation for individual-level clinical prognostication.

Our study found that glioma patients in more advanced stages of cancer experienced more aggressive cancer-related fatigue. As cancer progresses to later stages, the tumor burden increases, leading to more extensive physical changes and metabolic alterations (23). Patients may encounter systemic symptoms such as pain, weight loss, and anemia, alongside undergoing more aggressive treatments (24). The cumulative effect of treatments, combined with the disease itself, can lead to emotional distress that compounds feelings of exhaustion (25). In addition, the glioma patients with later stages may struggle to maintain proper nutrition due to factors such as nausea, loss of appetite, or gastrointestinal issues. These nutritional deficiencies may contribute to decreased energy levels and heightened fatigue in glioma patients. Mustian KM et al. analyzed 113 studies involving a sample of 11,525 cancer patients and also found that cancer stage (nonmetastatic, metastatic, or mixed) was an independent variable influencing the treatment effectiveness of cancer-related fatigue (26). Consequently, it is essential to provide enhanced attention and psychological support to patients with advanced glioma (27). This approach not only addresses their emotional and mental well-being but also plays a significant role in mitigating cancer-related fatigue.

Our study revealed that the score of Perceived Social Support Scale was an independent risk factor for cancer-related fatigue in glioma patients. The patient with a strong perception of social support was less likely to experience cancer-related fatigue. This is in line with the conclusion drawn by Zhihui Gu et al. in their study of patients with cervical cancers (6). The perception of social support refers to an individual’s belief or feeling about the availability and adequacy of support from their social network, which includes family, friends, colleagues, and community members. Studies have suggested that cancer patients who perceive a consistent availability of social support were more likely to report higher levels of resilience and lower levels of distress, regardless of the type of cancer they have (28). This may be attributed to the social support derived from role relationships, which helps to stabilize and foster positive self-esteem and confidence, thereby enhancing patients’ ability to cope with stress and reducing cancer-related fatigue (29). Therefore, seeking available social support or providing support to patients with glioma may facilitate reducing the incidence of fatigue.

Furthermore, we found that the quality of life plays a crucial role in the occurrence of cancer-related fatigue in patients with glioma. Patients with a higher quality of life typically enjoy better physical and mental health, which can help alleviate their perception and experience of fatigue (30). They are more motivated to adhere to treatment regimens and possess positive emotional states that enable them to cope effectively with the stressors associated with cancer and its treatment, potentially reducing feelings of fatigue (31). A recent multi-center randomized controlled trial found that the patient with high quality of life often maintain an active lifestyle and engage in regular physical activity, which can enhance energy levels and resilience against fatigue (32). A Systematic Review included 20 articles also indicated that supervised moderate-hard resistance training with or without moderate-vigorous aerobic exercise and dietary intervention may improve quality of life and alleviate cancer-related fatigue in cancer patients (33).

Nevertheless, there were several limitations in the present study. Firstly, the small sample size may have restricted the ability to identify significant associations between risk factors and the occurrence of cancer-related fatigue. Consequently, larger prospective cohort studies are needed to explore this topic further. Secondly, it is necessary to conduct additional prospective validation studies from multiple centers to enhance the reliability of our findings. Thirdly, this study is limited by the lack of a formal pre-study sample size calculation, which may reduce confidence in the statistical power. Last but not least, the follow-up period in this study was limited to just one month. A Longer follow-up is required to assess more distant outcomes.

## Conclusion

In conclusion, this study identified glioma cancer stage, perceived social support, and patient quality of life as independent risk factors for cancer-related fatigue. We developed and validated a nomogram that can noninvasively predict cancer-related fatigue in glioma patients. It would enable earlier detection and intervention for cancer-related fatigue, helping healthcare providers identify at-risk patients sooner. By facilitating timely interventions, it can improve the quality of life for glioma patients, ultimately enhancing their well-being and treatment outcomes.

## Data Availability

The original contributions presented in the study are included in the article/supplementary material. Further inquiries can be directed to the corresponding author.
